# Short-term auricular electrical stimulation rapidly elevated cortical blood flow and promoted the expression of nicotinic acetylcholine receptor α4 in the 2 vessel occlusion rats model

**DOI:** 10.1186/s12929-019-0526-9

**Published:** 2019-05-11

**Authors:** Tai-Hsiang Huang, Yi-Wen Lin, Chun-Ping Huang, Jing-Ming Chen, Ching-Liang Hsieh

**Affiliations:** 1 Graduate Institute of Acupuncture Science, College of Chinese Medicine, China Medical University, Taichung, 40402 Taiwan; 2 Chinese Medicine Research Center, China Medical University, Taichung, 40402 Taiwan; 3 Graduate Institute of Applied Science and Engineering, Fu Jen Catholic University, New Taipei City, 510 Taiwan; 4 Graduate Institute of Integrated Medicine, College of Chinese Medicine, China Medical University, 91 Hsueh-Shih Road, Taichung, 40402 Taiwan; 5 Department of Chinese Medicine, China Medical University Hospital, Taichung, 40447 Taiwan; 6 Research Center for Chinese Medicine and Acupuncture, China Medical Univeristy, Taichung, 40402 Taiwan

**Keywords:** Dementia, Bilateral carotid artery occlusion, Auricular electrical stimulation, Cerebral blood flow, Acetylcholine receptor

## Abstract

**Background:**

Vascular dementia is the second dementing illness after Alzheimer’s disease and caused by reduced blood flow to the brain, and affects cognitive abilities. Our previous study found that auricular electrical stimulation (ES) improved motor and learning impairment, and this phenomenon related with nicotinic acetylcholine receptor (nAChR) expressed cells. However, the underlying mechanism was not clear. In the present study, we investigated the effects of auricular ES on cortical blood flow (CBF) and acetylcholine (ACh) - nAChRs expressed cells.

**Methods:**

Vascular dementia rat animal model was established by permanent occlusions of common carotid arteries with 6–0 nylon suture filament. At 21 day after surgery, motor impairment was confirmed by rotarod test. 15-Hz auricular ES were applied to the ears for 20 min and CBF was recorded at the mean time. The brains were immediately dissected for immunohistochemical stain and western blot analysis.

**Results:**

Our results showed that 15-Hz auricular ES rapidly elevated CBF in the middle cerebral artery. The numbers of nAChR α4 immuno-positive cells and western blot levels were significally increased by 15-Hz auricular ES in the hippocampal CA2 output cortex. The numbers of choline acetyltransferase (ChAT) – a key enzyme for biosynthesis of ACh – immuno-positive cells and western blot levels had no significant differences.

**Conclusions:**

The present data suggested that the 15-Hz auricular ES for 20 min rapidly elevated cortical blood flow, promoted the expression of nAChR α4, and would be beneficial for the treatment of Alzheimer type and vascular type dementia.

## Background

Vascular dementia is the second dementing illness after Alzheimer’s disease. It is a progressive disease caused by reduced blood flow to the brain, and affects cognitive abilities such as loss of executive functions [27]. The neuropathological causes of vascular dementia include: cerebral small vessel disease, large vessel disease, strategic infarct, severe hypoperfusion state, angiopathy, haemorrhage/microbleed and hereditary vasculopathy [13]. Subcortical ischaemic vascular dementia, dues to small vessel disease and hypoperfusion, attributes to major proportion of them. Small vessel cerebral ischaemia causes neural damage to the hippocampus, the cerebral cortex, and the white matter areas [35]. The most common model is bilateral carotid artery occlusion (2 vessel occlusion, 2VO) in rats, leading to global chronic hypoperfusion and white matter injury [35].

The expression of nicotinic acetylcholine receptor (nAChR) declines with age and in dementia [15, 26, 31]. The functioning of AChR exerts neuroprotective effects against neurodegenerative diseases and prevents cognitive impairment. Cholinergic dysfunction is observed in patients with vascular dementia [22, 29] and animal models [4, 30]. Recent studies [9, 11] have reported that auricular acupuncture increases parasympathetic activity which then activates the solitary tract nucleus and has been demonstrated increasing the activity of vagus nerve [11, 18], but less understanding about cerebral hypoperfusion. In our previous study, the results found that auricular electrical stimulation (ES) ameliorated learning and memory impairment and has neuroprotective effects, which are related with nAChR expressed cells [17]. Therefore, we further investigated the effects of auricular ES to cortical blood flow (CBF) during stimulating phase and ACh – nAChRs expressed cells.

In this study, we first established a subcortical ischaemia 2VO rat model and investigated the effects of auricular ES to CBF by using laser-Doppler flowmeter. The nAChRs and choline acetyltransferase (ChAT) in hippocampus CA2 output cortex or habenular nuclei and were examined by immunohistochemical staining and western blot analysis. Our data suggested that 15-Hz auricular ES could elevate CBF and increase immunoreactive cells and western blot levels of nAChR α4.

## Methods

### Animals

The male Wistar rats weighing 250–300 g were used in this study and purchased (BioLASCO, Taipei, Taiwan). A 12–12-h light–dark cycle was maintained, and the room temperature was controlled at 25 °C. Adequate food and water were provided. The Animal Care and Use Committee of China Medical University approved the use of these animals. In addition, all procedures were performed according to the Guide for the Use of Laboratory Animals (National Academy Press).

### Induction of 2 VO animal model

The rats were anesthetized with chloral hydrate (400 mg/kg) (Sigma, St. Louis, MO, USA). Through a midline cervical incision, both common carotid arteries were exposed and permanent occlusion by 6–0 nylon suture filament. The surgical sites were sutured with 3–0 nylon filament suture and the rats were housed for 21 days.

### Grouping

The total of eighteen rats were randomly divided into three groups, each group was 6 rats as follows: (1) 2VO + 15-Hz ES group: in which the rats received 2 VO surgery and 15 Hz ES at right lateral ear (using clip electrodes with the cathode placed at the ear apex and anode at the ear lobe; 2 mA in intensity, 15 Hz for 20 min); (2) 2VO + Sham ES group: in which the rats received 2 VO surgery but not undergo ES; (3) Control group: in which the rats’ common carotid arteries were exposed without occlusion and received 15- Hz ES at right lateral ear.

### Rotarod test

Before 2VO surgery, rats were placed on a Rotamex (Columbus Instrument, Ohio, USA) with an initial speed of 4 rpm and which increased by 1 rpm every 8 s until the maximum speed of 40 rpm was attained. The latency spent by the rat on the rotarod before stepping out was recorded, the test was performed six times, and the average of the three longest times recorded was calculated as described previously [17]. At 21 day after surgery, rats were examined again.

### Measurement of CBF

After rotarod test, rats were anesthetized with chloral hydrate (400 mg/kg) and then placed in a stereotaxic apparatus in the prone position. The parietal bone was thinned using a grinding machine to remove skull. A laser Doppler Blood-Flow Monitor probe (DRT4; Moor Instrument Ltd., England) was placed 5 mm lateral, 1 mm posterior to the bregma to measure the CBF of middle cerebral artery - branch of bilateral carotid artery in the neocortex as described previously [17]. CBF was recorded in BPU (Blood Perfusion Units) within 20 min in the Pre-ES, ES and Post-ES phase, whether receiving auricular ES or not.

### Immunohistochemical staining

After measuring of CBF, rats were further anesthetized with overdose chloral hydrate, perfused with 200 mL of 0.9% saline, and then brains were removed. The brains were fixed in 4% paraformaldehyde for 3 days and were transferred to 30% sucrose (w/v) for 3 days. The brains were embedded in frozen section media (Leica Surgipath, USA) and cut into 15-μm sections in cryostat (Leica, USA), rinsed with 0.01% Tween 20 / phosphate buffered saline (PBS-T) twice and soaked in 3% H_2_O_2_ / methanol for 15 min to inhibit endogenous peroxidase activity. The sections were then blocking with 10% normal goat serum (Genemed Biotechnologies, CA, USA) for 20 min at room temperature. The sections were incubated with a primary antibody, the nAChR α4 (1:500) (abcam, MA, USA) or ChAT (1:500) (Thermo Scientific, MA, USA), at 4 °C overnight in a moisture chamber. The sections were subsequently incubated with the biotinylated-conjugated secondary antibody (Genemed Biotechnologies, CA, USA) for 10 min at room temperature, followed by incubation with the streptavidin-peroxidase complex (Genemed Biotechnologies, CA, USA). The sections were visualized using 3,3′-diaminobenzidine (Scytek Laboratories, UT, USA) as the chromogen and counterstained with hematoxylin(Genemed Biotechnologies, CA, USA). During the incubation steps, the sections were washed with PBS-T three times for 10 min per cycle. The stained sections were mounted in mounting media (Assistant-Histokitt, Germany), immunoreactive cells were calculated and photography was captured under the microscope (Axioskop 40, Zeiss, Germany).

### Western blot analysis

In addition, total of nine rats were randomly divided into three groups, each group was 3 rats as mentid habenular nucleus were excised, respectively, immediately for protein extraction. Total protein was prepared by homogenizing the cortex and the habenular nucleus in a lysis buffer. From each sample, 20 μg of proteins were extracted and analyzed through a BCA protein assay. They were subjected to 10% SDS-Tris glycine gel electrophoresis and transferred to a nitrocellulose membrane. The membrane was blocked with 5% nonfat milk in a TBST buffer (10 mmol/L of Tris, pH 7.5, 100 mmol/L of NaCl, and 0.1% Tween 20), incubated with a primary antibody, the nAChRα4 (1:1000,abcam,MA, USA) or ChAT (1:1000, Thermo Scientific, MA, USA) in PBS for overnight at 4 °C. Peroxidase-conjugated secondary antibody (1:2000) was used as the secondary antibody. The membrane was developed using the ECL-Plus protein detection kit.

### Statistical analysis

All data were presented as mean ± standard deviation. Statistical significance was analyzed through one-way ANOVA, followed by Tukey’s post hoc test. A *p* value of < 0.05 was considered statistically significant.

## Results

### 2VO animal model and auricular ES increased CBF

**Fig. 1:**
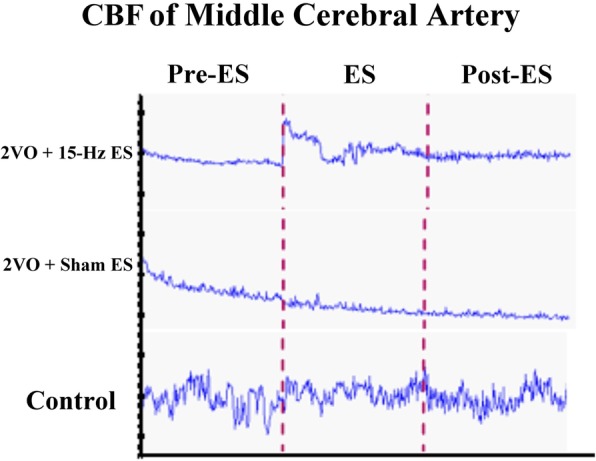
CBF were measured in Pre-ES, ES and Post-ES phase. After rotarod test, a laser Doppler Blood-Flow Monitor probe was put on rats’ middle cerebral artery under anesthetic condition and CBF were recorded within 20 min as showed

We estimated the motor function of 2VO animal model by rotarod test. The time of latency in the rotarod test before 2VO surgery had no significant differences among all groups (Table 1, Pre-2VO). At 21 day after surgery, the time of latency was 197.0 ± 56.7 (s) in 2VO + 15-Hz ES group, 147.3 ± 32.5 (s) in 2VO + Sham ES group, and 280.4 ± 63.7 (s) in Control group. The rats received 2 VO surgery had motor function impairment (Pre-2VO vs. Post-2VO in surgery groups, ***p < 0.001; *n* = 6; Table 1). We further investigated the effect of auricular ES to CBF value in Pre-ES, ES and Post-ES phase (Fig. 1a). After 2VO surgery, CBF was significantly reduced comparing to control group (124.7 ± 44.5 vs. 242.8 ± 107.2 BPU, *p < 0.05; *n* = 6; Table 2) in the Pre-ES phase. During ES phase, auricular ES significantly elevated CBF (19.4 ± 8.4 BPU, #*p* < 0.05; ES vs. Pre-ES phase; *n* = 6; Table 2) in the 2VO + 15 Hz ES group, but had no effects in the sham ES and control group. After auricular ES, all groups had no significant between Post-ES and ES or Pre-ES phase (Table 2).

**Table 1:** Latency to step out in the rotarod test. The eighteen Wistar rats were randomly divided into three groups, and time of latency to step out was recorded (s) before 2VO surgery (Pre-2VO). At 21 day after surgery, time of latency to step out was recorded among all groups

Group	Pre-2VO (s)	Post-2VO (s)
2VO + 15-Hz ES	380.1 ± 101.9	197.0 ± 56.7^***^
2VO + Sham ES	303.3 ± 43.6	147.3 ± 32.5^***^
Control	344.2 ± 82.7	280.4 ± 63.7

Data were represent as mean ± SD (s); *n* = 6; ^***^P < 0.001 Pre-2VO vs. Post-2VO

**Table 2:** CBF was recorded in the Pre-ES, ES and Post-ES phase. CSF was presented as average within 20 min. The deviation of different phase was calculated at EA vs Pre-ES, Post-ES vs ES, and Post-ES vs Pre-ES column

	2VO + 15-Hz ES	2VO + Sham ES	Control
Pre-ES	124.7 ± 44.5^*^	167.1 ± 48.8	242.8 ± 107.2
ES	144.1 ± 47.7	148.0 ± 51.0	235.1 ± 113.8
Post-ES	134.5 ± 53.8	136.2 ± 45.9	218.5 ± 110.2
EA vs Pre-ES	19.4 ± 8.4^#^	− 19.1 ± 30.3	−7.7 ± 27.7
Post-ES vs ES	−9.5 ± 11.4	−11.8 ± 21.5	− 16.7 ± 24.8
Post-ES vs Pre-ES	9.9 ± 17.9	−30.9 ± 45.4	− 24.4 ± 49.8

Data represent mean ± SD (*n* = 6). ^*^p < 0.05, 2VO + 15-Hz ES group vs Control group in Pre-ES phase. ^#^*P* < 0.05, EA vs Pre-ES phase in the 2VO + 15-Hz ES group

### Auricular ES promoted the expression of nAChR α4 in the hippocampal CA2 output cortex and habenular nuclei

**Fig. 2:**
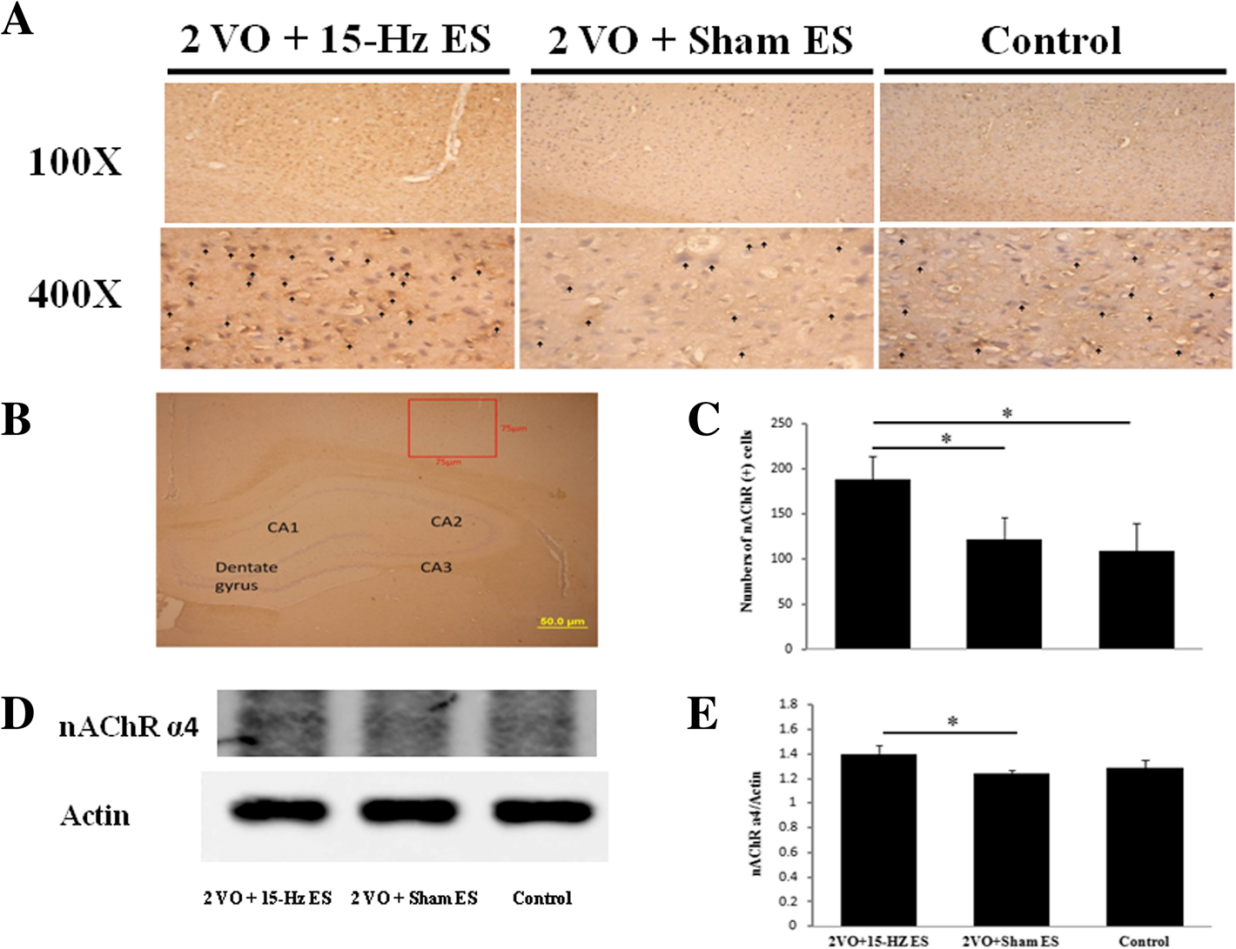
The immunohistochemical staining of the nAChR α4 in the hippocampal CA2 output cortex. The nAChR α4 immunoreactive cells were marked by arrowhead **a** in 400X and the counts of nAChR α4 immunoreactive cells were increased in 2VO + 15-Hz ES group; **b** nAChR α4 subtype was recognized in the hippocampal CA2 output cortex. (75 X 75 μm, scale bar = 50 μm); **c** Data represent mean ± SD in the counts of nAChR α4 immunoreactive cells (*n* = 6); **d** The levels of nAChR α4 were increased in western blot analysis in 2VO + 15-Hz ES group; **e** Data represent mean ± SD in the western blot levels of nAChR α4 (*n* = 3); *P < 0.05

**Fig. 3:**
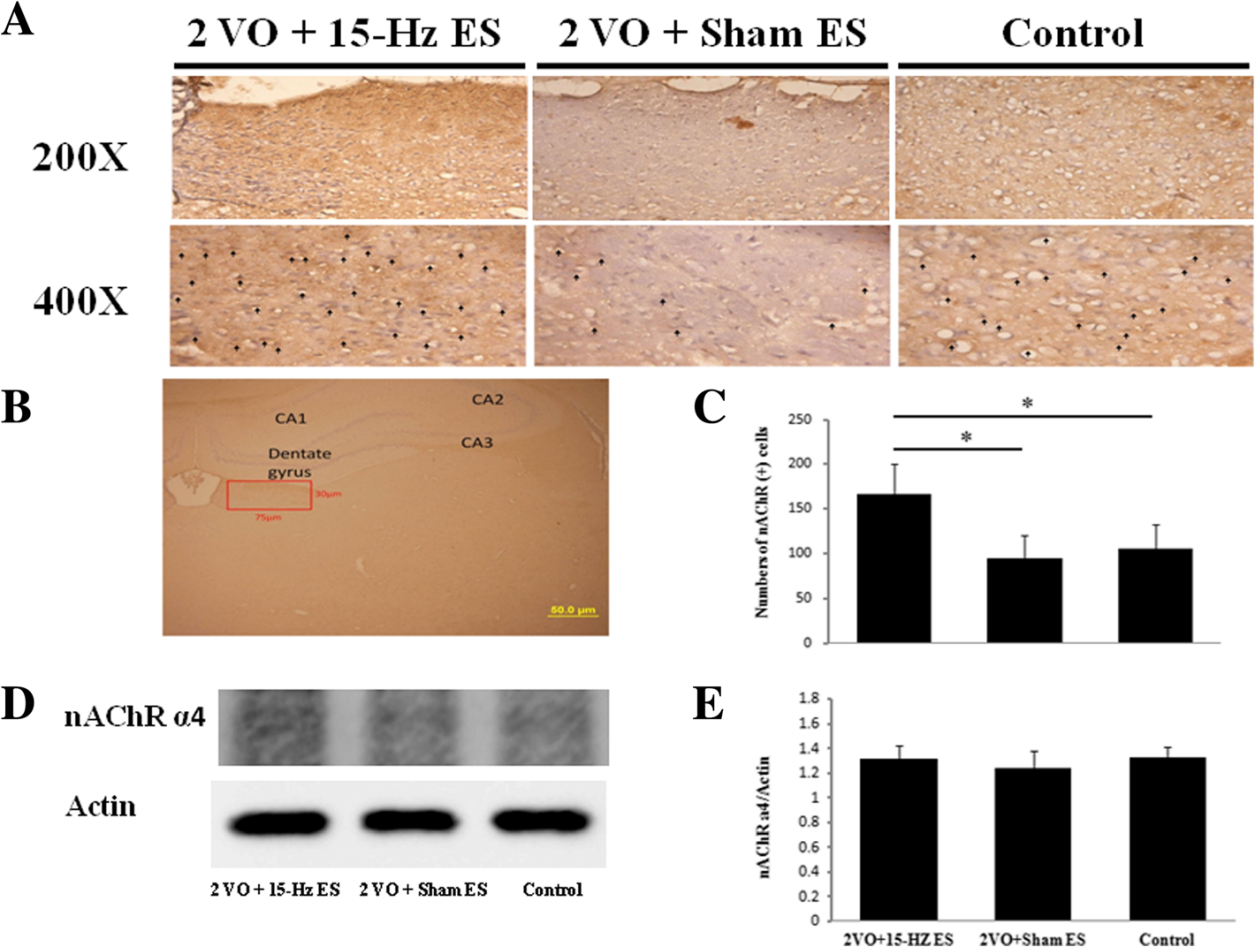
The immunohistochemical staining of nAChR α4 in the habenular nuclei. The nAChR immunoreactive cells were marked by arrowhead **a** in 400X and the counts of nAChR immunoreactive cells were increased in 2VO + 15-Hz ES group; **b** nAChR α4 subtype was recognized in the habenular nuclei. (75 X 30 μm, scale bar = 50 μm); **c** Data represent mean ± SD in the nAChR immunoreactive cells (*n* = 6); **d** The levels of nAChR α4 in western blot analysis; **e** Data represent mean ± SD in the western blot levels of nAChR α4 (*n* = 3); *P < 0.05

The nAChRs play a crucial role in the vasodilation mediated by nitric oxide in the cerebral cortex. These effects were dependent on increasing numbers of nAChR α4-like subtype [21, 33]. After measurement of CBF, the rat brains were immediately dissected, the nAChR α4 subtype was further recognized by immunohistochemical stain in the hippocampal CA2 output cortex (Fig. 2a and b) and habenular nuclei (Fig. 3a and b). Our results demonstrated auricular ES elevated the numbers of nAChR α4 subtype immuno-positive cells (188 ± 26, Fig. 2c; *n* = 6) compared to Sham ES (121 ± 25, *P < 0.05; *n* = 6) and control (109 ± 30, *P < 0.05; n = 6) in the hippocampal CA2 output cortex. It was also increased in 2VO + 15-Hz ES group (166 ± 35, Fig. 3c; *n* = 6), compared to Sham ES (95 ± 25, *P < 0.05; *n* = 6) and control (105 ± 28, *P < 0.05; *n* = 6) in habenular nuclei.

In the western blot analysis, the nAChR α4/actin ratio was 1.40 ± 0.07 (*n* = 3) in 2VO + 15-Hz ES group compare to Sham ES (1.24 ± 0.03, *P < 0.05; *n* = 3) and control (1.29 ± 0.06, *P* > 0.05; *n* = 3) in the hippocampal CA2 output cortex (Fig. 2d and e); the nAChR α4/actin ratio was 1.32 ± 0.10 (*n* = 3) in 2VO + 15-Hz ES group compare to Sham ES (1.24 ± 0.14, *P* > 0.05; *n* = 3) and control (1.33 ± 0.08, *P* > 0.05; *n* = 3) in the habenular nucleus (Fig. 3d and e) Our results demonstrated auricular ES elevated the levels of nAChR α4 in in the hippocampal CA2 output cortex, but not in the habenular nucleus..

### Auricular ES did not change the expression of ChAT in the hippocampal CA2 output cortex and habenular nuclei

**Fig. 4:**
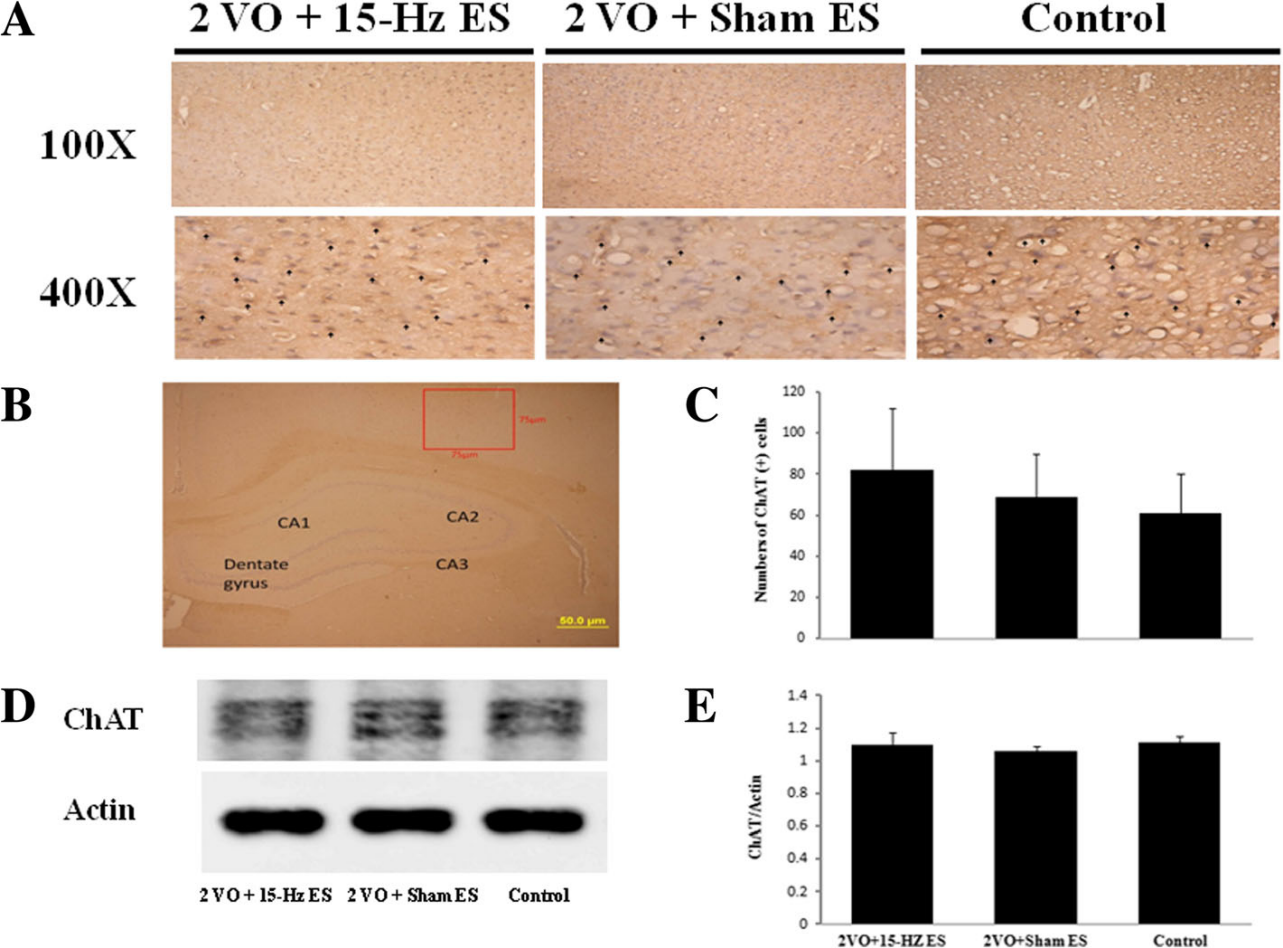
The immunohistochemical staining of the ChAT in the hippocampal CA2 output cortex. The ChAT immunoreactive cells were marked by arrowhead **a** in 400X and the counts of ChAT immunoreactive cells had no significant difference in each group; **b** ChAT was recognized in the hippocampal CA2 output cortex. (75 X 75 μm, scale bar = 50 μm); **c** Data represent mean ± SD in the counts of ChAT immunoreactive cells (*n* = 6); **d** The levels of ChAT in western blot analysis; **e** Data represent mean ± SD in the western blot levels of ChAT (*n* = 3)

**Fig. 5:**
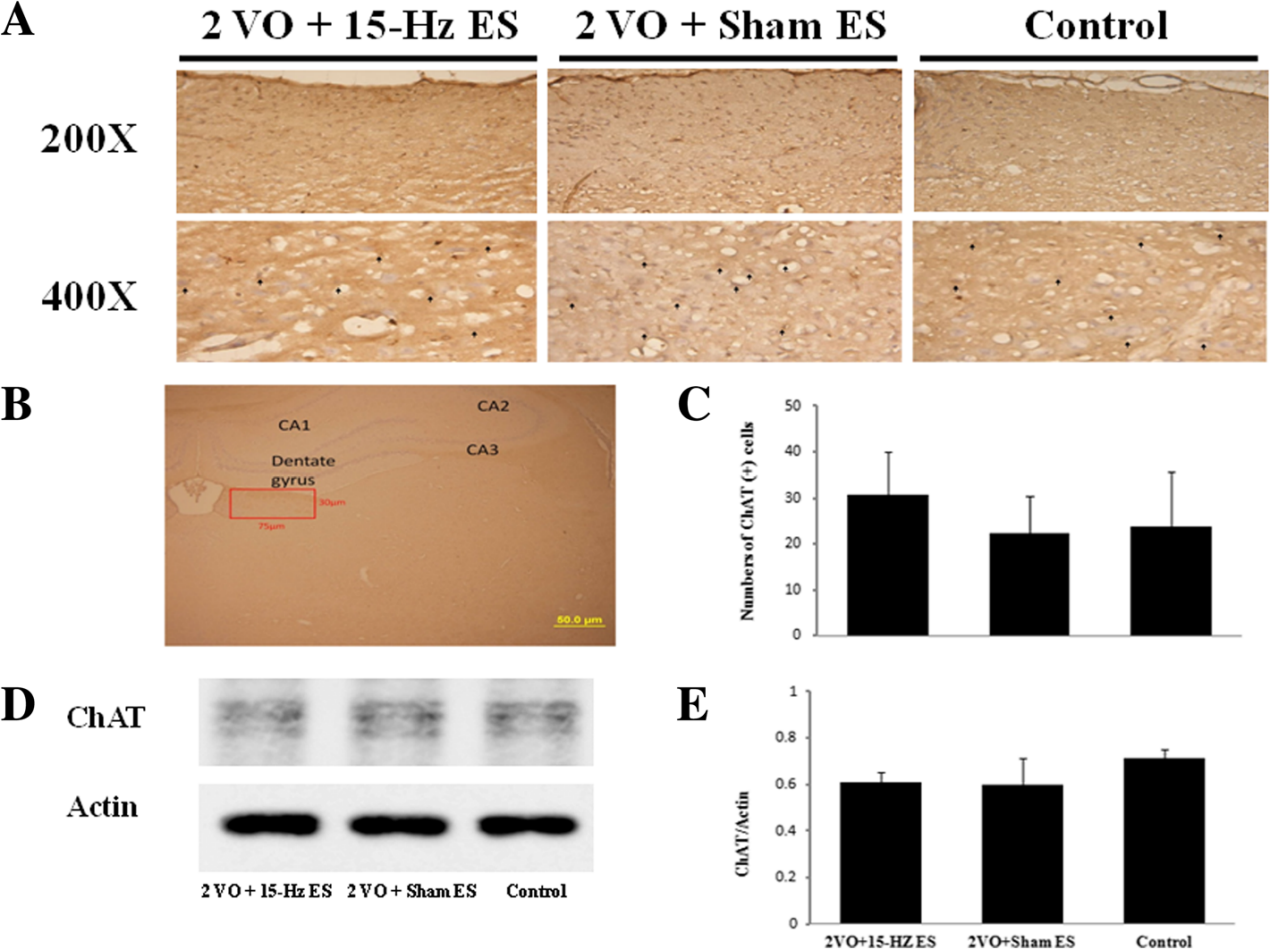
The immunohistochemical staining of ChAT in habenular nuclei. The ChAT immunoreactive cells were marked by arrowhead **a** in 400X and the counts of ChAT immunoreactive cells had no significant difference in each group; **b** ChAT was recognized in the habenular nuclei. (75 X 30 μm, scale bar = 50 μm); **c** Data represent mean ± SD in the ChAT immunoreactive cells (*n* = 6); **d** The levels of ChAT in western blot analysis; **e** Data represent mean ± SD in the western blot levels of ChAT (*n* = 3)

ChAT is a key enzyme for biosynthesis of ACh and as a specific indicator for monitoring the functional state of cholinergic neurons [25]. We further investigated ChAT immuno-positive cells in CA2 output cortex (Fig. 4a and b) and habenular nuclei (Fig. 5a and b). The ChAT immuno-positive cells was 82.17 ± 30.23 (*n* = 6) in 2VO + 15-Hz ES group compare to Sham ES (68.83 ± 21.18, *P* > 0.05; *n* = 6) and control (60.83 ± 19.67, *P* > 0.05; *n* = 6) in the hippocampal CA2 output cortex (Fig. 4c); the ChAT immuno-positive cells was 30.83 ± 9.20 (*n* = 6) in 2VO + 15-Hz ES group compare to Sham ES (22.33 ± 8.04, *P* > 0.05; *n* = 6) and control (23.67 ± 12.13, *P* > 0.05; *n* = 6) in the habenular nucleus (Fig. 5c).

In the western blot analysis, the ChAT /actin ratio was 1.10 ± 0.07 (*n* = 3) in 2VO + 15-Hz ES group compare to Sham ES (1.06 ± 0.03, *P* > 0.05; *n* = 3) and control (1.11 ± 0.04, *P* > 0.05; *n* = 3) in the hippocampal CA2 output cortex (Fig. 4d and e); the ChAT/actin ratio was 0.61 ± 0.04 (*n* = 3) in 2VO + 15-Hz ES group compare to Sham ES (0.60 ± 0.11, *P* > 0.05; *n* = 3) and control (0.71 ± 0.04, *P* > 0.05; *n* = 3) in the in the habenular nucleus (Fig. 5d and e). Our results showed short-term 15-Hz auricular ES did not change the expression of ChAT in the hippocampal CA2 output cortex and habenular nuclei.

## Discussion

CBF has been predicted as a marker for the progression from mild cognitive impairment into Alzheimer’s disease. The reconstruction of global chronic hypoperfusion animal models seems to be an ideal strategy to elucidate the relationship. Here, we showed 15-Hz auricular ES increased CBF in real-time by monitoring the middle cerebral artery in 2 VO rats model. This finding provided direct evidence for auricular acupuncture in the treatment of dementia. The increase of CBF mediated by acupuncture is believed as a reflex response through ACh originating in the nucleus of basalis of Meynert [34] and vasodilation by endothelial nitric oxide synthase [16].

Cholinergic therapies improve cognitive impairment in dementia patients [14, 23, 24] and the mechanisms of cholinergic treatment are not still well-known, may act through the cholinergic anti-inflammatory pathway, regulation of oxidative stress and energy metabolism [36]. The decrease of nicotinic receptor has been demonstrated in patients with Alzheimer disease [37] and it suggests cholinergic receptors as candidates to rescue dementia. In the present data, we found 15-Hz auricular ES promoted the expression of nAChR α4 subtypes in the hippocampus CA2 output cortex. It is now well known that exposure to nicotine results in nAChR up-regulation in cultured cells [2, 8], animals [39] and humans [5, 6]. The augmentation of nAChRs may be a positive feedback process through ACh release [1].

The nicotine-induced nAChR α4β2 up-regulation has showed neuroprotection against excitotoxicity [32, 38]. This mechanism may be involved in survival, cell proliferation and anti-apoptosis pathway [19, 28]. However, nAChR α4β2 has anti-inflammatory effects [12] or inflammatory reaction [20] oppositely. It may be induced by expression of nAChR α4β2 in different brain regions and different cell types. The cholinergic neurons, which originate from basal forebrain and medial septal region, innervate to cortical mantle, olfactory bulb, hippocampus and amygdala. ChAT is synthesized in the perikayon of cholinergic neurons and transported to the nerve terminals, controlled by both slow (1–23 mm/day) and rapid (30–200 mm/day) axoplasmic flows [25]. In the present data, we found short-term (once, 20 min) 15-Hz auricular ES did not increase the expression of ChAT. This phenomenon may have been due to the transport rate of ChAT. Taken together, 15-Hz auricular ES elevated CBF and nAChR α4 levels in the hippocampal CA2 output cortex in the 2 VO animal model, suggesting 15-Hz auricular ES is beneficial for the treatment of Alzheimer type and vascular type dementia. Auricular stimulation can through auriculo-vagal afferent pathway to nucleus tractus solitaris (NTS). The NTS plays an important role in the regulation of autonomic activities and project widely the information that receives from other brain structure [10]. Ear lobe ES can induce the central projection of the auricular branch of the vagus nerve to NTS in human [7]. In addition, anticonvulsive effect of electroacupuncture results from the collaboration of its anti-inflammatory and neurotrophic action through NTS in epilepsy models [3]. Therefore, suggesting NTS plays a critical role in 15-Hz auricular ES elevated CBF and nAChR α4, this suggestion need further study in the future.

## Conclusions

In conclusion, the 15-Hz auricular ES for 20 min led to elevated CBF of middle cerebral artery, increase the expression of nAChR in the cerebral cortex. Our present data provide evidences auricular ES would be useful for the treatment of Alzheimer type and vascular type dementia.

## Abbreviations

2VO: 2 vessel occlusion
CBF: Cortical blood flow
ChAT: Choline acetyltransferase
ES: Electrical stimulation
nAChR: Nicotinic acetylcholine receptor
NTS: Nucleus tractus solitari
